# The Impact of Stability Considerations on Genetic Fine-Mapping

**DOI:** 10.1101/2023.04.11.536456

**Published:** 2023-04-13

**Authors:** Alan Aw, Lionel Chentian Jin, Nilah Ioannidis, Yun S. Song

**Affiliations:** 1 Department of Statistics, University of California, Berkeley; 2 Center for Computational Biology, University of California, Berkeley; 3 McKinsey & Company, Seattle; 4 Computer Science Division, University of California, Berkeley

## Abstract

Fine-mapping methods, which aim to identify genetic variants responsible for complex traits following genetic association studies, typically assume that sufficient adjustments for confounding within the association study cohort have been made, e.g., through regressing out the top principal components (i.e., residualization). Despite its widespread use, however, residualization may not completely remove all sources of confounding. Here, we propose a complementary stability-guided approach that does not rely on residualization, which identifies consistently fine-mapped variants across different genetic backgrounds or environments. We demonstrate the utility of this approach by applying it to fine-map eQTLs in the GEUVADIS data. Using 378 different functional annotations of the human genome, including recent deep learning-based annotations (e.g., Enformer), we compare enrichments of these annotations among variants for which the stability and traditional residualization-based fine-mapping approaches agree against those for which they disagree, and find that the stability approach enhances the power of traditional fine-mapping methods in identifying variants with functional impact. Finally, in cases where the two approaches report distinct variants, our approach identifies variants comparably enriched for functional annotations. Our findings suggest that the stability principle, as a conceptually simple device, complements existing approaches to fine-mapping, reinforcing recent advocacy of evaluating cross-population and cross-environment portability of biological findings. To support visualization and interpretation of our results, we provide a Shiny app, available at: https://alan-aw.shinyapps.io/stability_v0/.

## 1 Introduction

An important challenge faced by computational precision health research is the lack of generalizability of biological findings, which are often obtained by studying cohorts that do not include particular communities of individuals. Known as the *cross-population generalizability* or *portability problem*, the challenge persists in multiple settings, including gene expression prediction (Keys et al. 2020) and polygenic risk score prediction (Mostafavi et al. 2020). Generalizable biological signals, such as the functional impact of a variant, are important, as they ensure that general conclusions drawn from cohort-specific analyses are not based on spurious discoveries. Portability problems are potentially attributable to cohort-biased discoveries being treated as generalizable true signals. Efforts to address such problems have included the use of diverse cohorts typically representing multiple population ancestries (Márquez-Luna et al. 2017), meta-analyses of earlier studies across diverse cohorts (Turley et al. 2021; Han and Eskin 2012; Morris 2011; Willer et al. 2010), or focusing on biological markers that are more likely *a priori* to play a causal role, e.g., rare variants (Zaidi and Mathieson 2020) or the transcriptome (Liang et al. 2022).

In this paper, we consider an approach based on the notion of *stability* to improve generalizability. Being a pillar of veridical data science (Yu and Kumbier 2020), stability refers to the robustness of statistical conclusions to *perturbations* of the data. Perturbations are not arbitrary, but instead they encode the practitioner’s beliefs about the quality of the data and the nature of relationship between variables. Well-chosen perturbation schemes will help the practitioner obtain statistical conclusions that are robust, in the sense that they are generalizable rather than spurious findings, and therefore more likely to capture the true signal. For example, in sparse linear models, prioritizing the stability of effect sizes to cross-validation “perturbations” leads to a much smaller set of selected features without reducing predictive performance (Lim and Yu 2016). In another example involving the application of random forests to detect higher-order interactions between gene regulation features (Basu et al. 2018), interactions stable to bootstrap perturbations are also largely consistent with known physical interactions between the associated transcription factor binding or enhancer sites.

To further investigate the utility of the stability approach, we focus on a procedure known as (genetic) fine-mapping (Schaid et al. 2018). Fine-mapping is the task of identifying genetic variants that causally affect some trait of interest. From the viewpoint of stability, previous works focusing on cross-population stability of fine-mapped variants typically use Bayesian linear models. The linear model has allowed modeling of heterogeneous effect sizes across different user-defined populations (e.g., ethnic groups, ancestrally distinct populations, or study cohorts), and it is common to assume that causal variants share correlated effects across populations (see, e.g., eq. (24) of LaPierre et al. 2021 or eq. (9) of Wen et al. 2015).

Unlike the parametric approaches described above, we here apply a *non-parametric* fine-mapping method to GEUVADIS (Lappalainen et al. 2013), a database of gene expression traits measured across individuals of diverse genetic ancestries and from different geographical environments, whose accompanying genotypes are available through the 1000 Genomes Project (1000 Genomes Project Consortium et al. 2015). We apply fine-mapping through two approaches, one that is commonly used in practice and another that is guided by stability. We evaluate the agreement of fine-mapped variants between the two approaches, and then measure the functional significance of variants picked by both approaches using a much wider range of functional annotations than considered in previous studies. By performing various statistical tests on the functional annotations, we evaluate the advantages brought about by the incorporation of stability. Finally, to support visualization of results at both the single gene and genome-wide levels, we provide an interactive Shiny app which is available at: https://alan-aw.shinyapps.io/stability_v0/. Our app is open-source and designed to support geneticists in interpreting our results, thereby also addressing a growing demand for accessible, *integrative* software to interpret genetic findings in the age of big data and variant annotation databases.

## 2 Results

### 2.1 Experimental Design

We apply the fine-mapping method PICS (Taylor et al. 2021; Farh et al. 2015; see Algorithm 1 in Subsection 4.1) to the GEUVADIS data (Lappalainen et al. 2013), which consists of T=22,720 gene expression traits measured across N=445 individuals, with their accompanying genotypes obtained from the 1000 Genomes Project (1000 Genomes Project Consortium et al. 2015). These individuals are of either European or African ancestry, with about four fifths of the cohort made up of individuals of (self-identified) European descent. In particular, these ancestrally different subpopulations have distinct linkage disequilibrium patterns and environmental exposures, which constitute potential confounders that we wish to stabilize the fine-mapping procedure against.

**Figure 1:**
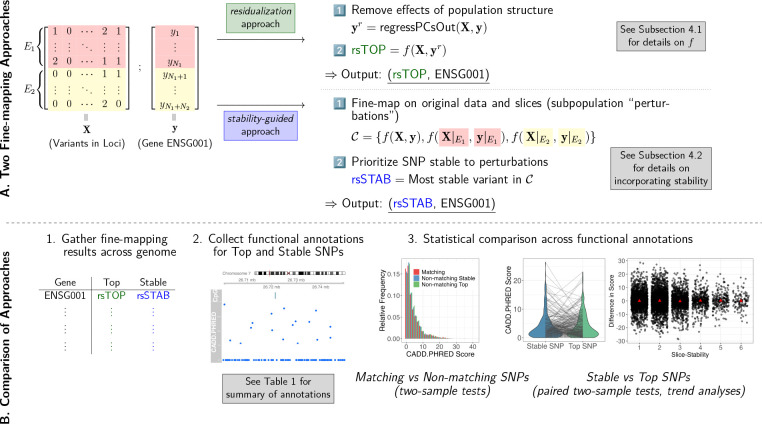
An overview of our study of the impact of stability considerations on genetic fine-mapping. **A.** The two ways in which we perform fine-mapping, the first of which (colored in green) prioritizes the stability of variant discoveries to subpopulation perturbations. The data illustrates the case where there are two distinct environments, or subpopulations (denoted *E*_1_ and *E*_2_), that split the observations. **B.** Key steps in our comparison of the stability-guided approach with the popular residualization approach.

PICS, like many eQTL analysis methods, requires the lead variant at a locus to compute posterior probabilities, so we perform marginal regressions of each gene expression trait against variants within the fine-mapping locus. Our implementation of PICS generates three sets of variants, which represent putatively causal variants that are (marginally) highly associated, moderately associated and weakly associated with the gene expression phenotype. (See Section 4, Materials and Methods for details.) We compare two versions of the fine-mapping procedure — one that is typically performed in practice, and another that is motivated by the stability approach. See Figure 1A.

#### Stable variant.

Specifically, to incorporate stability into fine-mapping and obtain what we call the *stable variant* or stable SNP, we encode into the algorithm our belief that for a gene expression trait, a causal variant would presumably act through the same mechanism regardless of the population from which they originate. Indeed, if a set of genetic variants were causal, the same algorithm should report it, if run on subsets of the data corresponding to heterogeneous populations. This belief is consistent with recent simulation studies showing that the inclusion of GWAS variants discovered in diverse populations both mitigates false discoveries driven by linkage disequilibrium differences (Li et al. 2022) and improves generalizability of polygenic score construction (Cavazos and Witte 2021). Hence, the stable variant is the variant with the highest probability of being causal, conditioned on being reported by PICS in the most number of subpopulations. Section 4.2 provides a formal definition of the stable variant.

#### Top variant.

The stability consideration we have described is closely related to a popular procedure known as correction for population structure, which residualizes the trait using measured confounders (e.g., top principal components computed from the genotype matrix). We call the variant returned by the residualization approach the *top variant* or top SNP. The residualization approach removes the effects of genetic ancestry and environental exposures on a trait, and is used to avoid the risk of low statistical power borne by stratified analyses. The top variant is formally defined towards the end of Section 4.1. We remark that the top variant is a function of the residualized trait, as opposed to the stable variant, which is a function of the unresidualized trait (see Figure 1A).

#### Comparison.

We compare the results obtained by using each approach, across a range of categories of functional annotations including conservation, pathogenicity, chromatin accessibility, transcription factor binding and histone modification, and across biological assays and computational predictions. See Table 1 for full list of annotations covered. (Supplementary Material Section S2 contains a full description of each quantity and its interpretation.)

Figure 1B summarizes the key steps of our investigation. To be clear, a first comparison is between the set of genes for which the top and stable variants match and the set of genes for which the top and stable variants disagree; comparisons are made between matching variants and one of the non-matching sets of variants (top or stable). A second comparison is restricted to the set of genes with non-matching top and stable variants, i.e., between *paired* sets of variants fine-mapped to a gene, where one set of a pair corresponds to the output of the residualization approach whereas the other corresponds to the output of the stability-guided approach. We additionally compare across three fine-mapping output sets, corresponding to variants that have a high, moderate or weak marginal association with the expression phenotype. For brevity, we term these three sets Potential Set 1, Potential Set 2 and Potential Set 3, respectively. (See Algorithm 1 for details.) For each Potential Set, we find the top and stable variants as described in Section 4.2.

### 2.2 Stability-guided variants frequently do not match residualization variants

**Figure 2:**
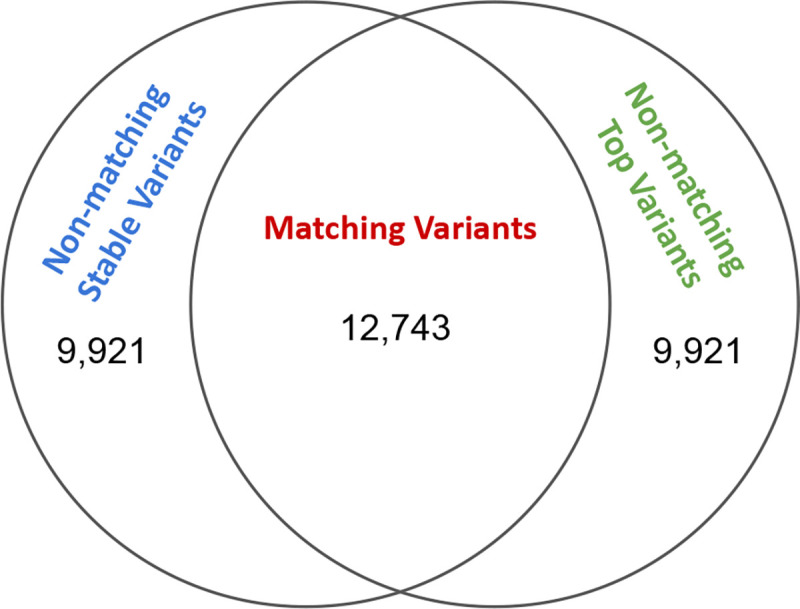
Venn diagram showing the number of matching and non-matching variants for Potential Set 1.

As a preliminary analysis, when interrogating if the residualization and stability-guided approaches produce the same candidate causal variants, we find that for Potential Set 1, which corresponds to the set typically reported in fine-mapping studies, 56.2% of genes had matching top and stable variants (see Figure 2). Moving down potential sets, we find less agreement (Potential Set 2: 36.2%, Potential Set 3: 25.6%), providing evidence that as the marginal association of a variant with the expression phenotype decreases, the two approaches prioritize different signals when searching for putatively causal variants.

For the rest of this Section, we report only results for Potential Set 1, given that it corresponds to the set typically reported in fine-mapping. Results for the other potential sets are provided in Supplementary Material Sections S4 and S5.

### 2.3 Matching versus Non-Matching Variants

For each gene, our algorithm finds the top eQTL variant and the stable eQTL variant, which may not coincide. We thus run (one-sided) unpaired Wilcoxon tests on matching and non-matching sets of variants, to detect significant functional enrichment of one set of variants over the other. We find that the top variants that are also stable for the corresponding gene $\left(N_{\text{match}}=12,743\right.$ maximum annotatable) score significantly higher in functional annotations than the top variants that are not stable $\left(N_{\text{non-match}}=9,921\right.$ maximum annotatable). Notably, 361 out of 378 functional annotations report one-sided greater *p*-values < 0.05 for the matching (i.e., both top and stable) variants after correcting for multiple testing using the Benjamini-Hochberg (BH) procedure, with many of these annotations measuring magnitudes of functional impact or functional enrichment (e.g., Enformer perturbation scores, FATHMM.XF score). Amongst the 17 remaining functional annotations, none has significantly lower scores for the matching variants. (Supplementary Material Section S4 lists these functional annotations in detail.) We also find that empirically, the matching variants tend to have greater agreement in posterior probabilities than non-matching variants (Supplementary Figures S1 and S2).

**Figure 3:**
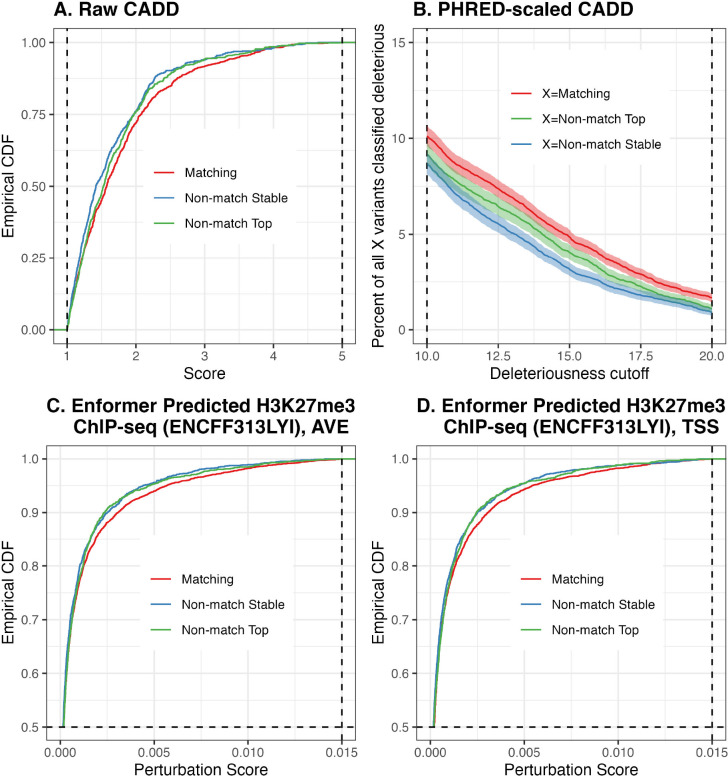
*Top Row.* CADD scores. **A.** Empirical cumulative distribution functions of raw CADD scores of matching and non-matching variants across all genes, for Potential Set 1. Non-matching variants are further divided into stable and top variants, with a score lower threshold of 1.0 and upper threshold of 5.0 used to improve visualization. **B.** For a deleteriousness cutoff, the percent of (i) all matching variants, (ii) all non-matching top variants, and (iii) all non-matching stable variants, which are classified as deleterious. We use a sliding cutoff threshold ranging from 10 to 20 as recommended by CADD authors. *Bottom Row.* Empirical cumulative distribution functions of perturbation scores of Enformer-predicted H3K27me3 ChIP-seq track. Score upper threshold of 0.015 and empirical CDF lower threshold of 0.5 used to improve visualization. **C.** Perturbation scores computed from predictions based on centering input sequences on the gene TSS as well as its two flanking positions. **D.** Perturbation scores computed from predictions based on centering input sequences on the gene TSS only.

As an example of a significant functional annotation, consider raw CADD scores (Rentzsch et al. 2019) — a higher value of which indicates a greater likelihood of deleterious effects. Out of the 22, 559 genes for which both the top and the stable variant are annotatable, looking at the distribution of top variant scores, the one corresponding to the 12, 685 genes with matching top and stable variants stochastically dominates the one corresponding to the remaining 9, 874 genes (Figure 3A). This relationship is more pronounced when we inspect PHRED-scaled CADD scores, where we apply a sliding cutoff threshold for calling variant deleteriousness (i.e., potential pathogenicity — see Rentzsch et al. 2019). We find that a greater proportion of matching variants than of either non-matching variant is classified as deleterious under the typical range of deleteriousness cutoffs (Figure 3B).

Another example demonstrating significant functional enrichment of matching variants over non-matching variants is the perturbation scores on H3K9me3 ChIP-seq peaks, as predicted by the Enformer (Avsec et al. 2021). Out of the 6, 364 genes for which the distance of both the top and the stable variant to the TSS are within the Enformer input sequence length constraint, looking at the the distribution of top variant scores, the one corresponding to the 4, 491 genes with matching top and stable variants stochastically dominates the one corresponding to the remaining 1, 873 genes (Figure 3C,D). This relationship is true regardless of whether perturbation scores are calculated from an average of input sequences centered on the gene TSS and its two flanking positions (Figure 3C), or from input sequences centered on the gene TSS only (Figure 3D). (See Supplementary Material Section S3 for details on Enformer annotation calculation.)

Similarly, amongst all stable variants, those variants that match the top variant are significantly more enriched in functional annotations: 363 functional annotations report one-sided greater BH-adjusted *p*-values < 0.05 for the matching variants, and none of the remaining 15 annotations present significant depletion for the matching variants set.

### 2.4 Top versus Stable variants when they do not match

**Figure 4:**
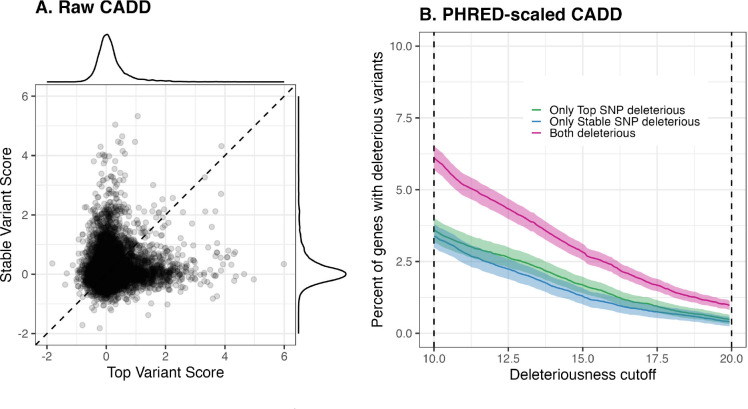
**A.** Paired scatterplot of raw CADD scores of both top and stable variant for each gene, for Potential Set 1. **B.** Percent of genes that are classified as (i) having deleterious top variant only, (ii) having deleterious stable variant only, and (iii) having both top and stable variant deleterious, using a sliding cutoff threshold ranging from 10 to 20 as recommended by CADD authors.

Focusing on the genes for which the top and stable variants are different, we run (one-sided) paired Wilcoxon tests to detect significant functional enrichment of one set of variants over the other. We find in general that for some genes stable variants can carry more functional impact than top variants, and for other genes top variants carry more functional impact — although neither of these patterns is statistically significant genome-wide after multiple testing correction using the BH procedure. For example, for raw CADD scores, out of the 9, 874 genes for which the top and the stable variants do not match, 4, 906 genes have higher scoring stable variants than top variants, whereas 4,968 genes have higher scoring top variants than stable variants (Figure 4A). Looking at the accompanying PHRED-scaled CADD scores (CADD PHRED) for Potential Set 1, when applying a sliding cutoff threshold for calling variant deleteriousness, we find that even though a higher fraction of genes have top but not stable variant classified as deleterious, the difference is usually not significant (Figure 4B). Figure 4B also demonstrates that no matter how the cutoff is chosen, there are genes for which the top variant is not classified deleterious while the stable variant is. Taken together, these observations suggest that the stability-guided approach can sometimes be more useful at identifying variants of functional significance, and in a broad sense both fine-mapped variants should be equally prioritized for potential of carrying functional impact.

Because our comparison between the top and stable variants yielded no significant functional enrichment of one over the other, we investigate whether external factors — for example, the posterior probability of the variants — might moderate the relative enrichment of one variant over the other (i.e., we perform *trend analysis* — see Section 4.3 for a list of all moderators). Here, we find that all but one of the moderators considered — namely, the Posterior Probability of Top Variant (see Table 2) — did not produce any significant trends for Potential Set 1. There is a small but significant positive correlation (Pearson’s r=0.05) between the posterior probability of the top variant and the difference between the stable variant and top variant FIRE scores (Ioannidis et al. 2017), and a small but significant negative correlation (Pearson’s r=−0.07) between the posterior probability of the top variant and the difference between the stable variant and top variant Absolute Distance to Canonical TSS. (Details are reported in Supplementary Material Section S5.)

#### Additional comparisons.

We perform various *conditional analyses* to evaluate whether additional restrictions to characteristics of fine-mapping outputs may boost the power of either approach over the other. Such characteristics include (i) the positive posterior probability support (i.e., how many variants reported a positive posterior probability from fine-mapping), and (ii) the posterior probability of the top or stable variant. Results from our conditional analysis of (i) are reported in Supplementary Material Section S6. As an example, for (ii) we further perform a comparison between top and stable variants, by restricting to genes where the posterior probability of the top variant or the stable variant exceeds 0.9. Such restriction of valid fine-mapped variant-gene pairs is useful in training variant effect prediction models requiring reliable positive and negative examples, as seen in Wang et al. (2021). We find that for genes where the top variant posterior probability exceeds 0.9, there is no significant enrichment of the top variant over the stable variant across the 378 annotations considered (all BH-adjusted *p*-values exceed 0.05). Interestingly, when focusing on genes where the stable variant posterior probability exceeds 0.9, FIRE scores of the top variant are significantly larger than the stable variant (BH-adjusted *p*-value = 1 × 10^−6^). Detailed results are reported in Supplementary Material Section S7.

## 3 Discussion

Our present work has shown that a stability-guided approach complements existing approaches to detect biologically meaningful variants in genetic fine-mapping. Through various statistical comparisons, we have found that prioritizing the agreement between existing approaches and a stability-guided approach enhances the functional impact of the fine-mapped variant. Incorporating stability into fine-mapping also provides an adjuvant approach that helps discover variants of potential functional impact in case standard approaches fail to pick up variants of functional significance. Our findings are consistent with earlier reports of stable discoveries having the tendency to capture actual physical or mechanistic relationships, potentially making such discoveries generalizable or portable (Basu et al. 2018).

The link between stability and generalizability is not new. In the machine learning and statistics literature, it has been shown that stable algorithms provably lead to generalization errors that are well-controlled (Bousquet and Elisseeff 2002, Theorem 17, p. 510). Furthermore, in certain classes of algorithms (e.g., sparse regression), it has been demonstrated empirically that this mathematical relationship is explained precisely by the stable algorithm removing spurious discoveries (Lim and Yu 2016).

The stability-guided approach is also distinct from other subsampling approaches, such as the bootstrap (Efron and Tibshirani 1994). First, the bootstrap is more often deployed as a method for calibrating uncertainty surrounding a prediction, which is not the objective of our method. Next, in settings where the bootstrap is deployed as a type of perturbation against which a prediction or estimand is expected to be stable (Basu et al. 2018), a stability threshold is implicitly needed and would require tuning to be chosen (e.g., in Basu et al. 2018 this threshold was chosen to be 50%). Here, we leverage interpretable existing external annotations to define the perturbation against which we expect the fine-mapped variant to be stable — in other words, the user relates what is meant by the fine-mapped variant being stable to a biologically meaningful concept like “portable across environmentally and LD pattern-wise heterogeneous populations.”

The last sentence in the preceding paragraph suggests there are teleological similarities between the stability-guided approach and meta-analysis approaches (Turley et al. 2021). We emphasize that meta-analyses rely on already analyzed cohorts, thereby implicitly assuming that *within-cohort* heterogeneities have been sufficiently accounted for prior to the reporting of findings for that cohort. The stability-guided approach, however, is relevant to the *cohort-specific analysis itself*, where existing approaches may present methodological insufficiencies resulting in inflated false discoveries.

In other words, whereas the goal of meta-analysis may be stated as identifying consistent hits across cohorts while also assuming that findings specific to each cohort are reliable, the goal of a stability-guided approach is to search for consistent signals despite the presence of potential confounders within a single cohort. Our analysis has focused on comparing the stability-guided approach against residualization, a convenient and popular approach to account for population structure, which reflects this difference in purpose.

In investigating the impact of stability considerations on the fine-mapping algorithm, our analyses integrated multiple families of functional annotations. These include biological assays and tracks (Ensembl gene regulatory regions, percent GC, etc.), classical variant interpretation scores and statistics (CADD, FIRE, etc.), and modern deep learning-based annotations (Enformer). We further summarize our findings using open-source interactive software, enabling the broader biological community to interpret our results across 378 annotations and all genes, or at the single gene level. Altogether, our work not only provides pristine ways to synthesize new findings from the expanding suite of in-silico mutagenesis models with biological quantities reported from mathematical models in biology and classical statistical principles, but also presents the results in a format accessible to biologists seeking to benefit from computational insights.

Our present work is not without limitations. First, we have chosen to focus on a non-parametric approach to fine-mapping, but multiple parametric fine-mapping approaches have been extended to incorporate cross-population heterogeneity (e.g., Wen et al. 2015; LaPierre et al. 2021; Lu et al. 2022). While our present work is a proof-of-concept of the applicability of the stability to genetic fine-mapping, we believe that future work focusing on comparing functional impact of variants prioritized by our stability-driven approach or these parametric methods will shed more light on the efficacy of the stability principle at detecting generalizable biological signals. Second, our analysis implicitly assumes that there is exactly one cis-eQTL that is prioritized by PICS. It is possible to modify the algorithm to return multiple variants, but this would require a more theoretical investigation, so we defer such technical explorations to future work. Third, while we have relied on numerous computational and experimental annotations to evaluate the functional impact of our fine-mapped variants, there is a possibility that some of the computationally predicted variant annotations (e.g., CADD, Enformer, FIRE) are themselves biased owing to the lack of diversity of training data. Although recent work has evaluated the performance of these computational annotation tools on different tissues and cell types, to our knowledge, there are currently no benchmarks for evaluating the performance of computational annotations on diverse human cohorts. We believe such studies will be important for ensuring that our findings support the theory that stable variants carry generalizable biological signal.

In closing, while our work explores stability to subpopulation perturbations where subpopulations are defined by ancestry, we emphasize that our stability-guided slicing methodology is applicable to all settings where meaningful external labels are available to the data analyst. For instance, environmental or geographical variables, which are well-recognized determinants of some health outcomes (Favé et al. 2018; Abdellaoui et al. 2022) and arguably better measure potential confounders than ancestral population labels, can be the basis on which slices are defined in the stability-guided approach. As the barriers to access larger biobank-scale datasets, which contain such aforementioned variables, continue to be lowered, we expect stability-driven analyses conducted on such data and relying on carefully defined slices will help users better understand genetic drivers of complex traits. Given its utility in our present work, we believe that the stability approach in precision medicine may find uses beyond genetic fine-mapping and few other biological tasks previously studied, ultimately empowering the discovery of veridical effects not previously known.

## 4 Materials and Methods

We use publicly available GEUVADIS B-lymphocyte RNA-seq measurements from 445 individuals (Lappalainen et al. 2013), whose genotypes are also available from the 1000 Genomes Project (1000 Genomes Project Consortium et al. 2015). The 445 individuals come from five populations: Tuscany Italian (TSI, N=91), Great British (GBR, N=86), Finnish (FIN, N=92), Utah White American (CEU, N=89) and Yoruban (YRI, N=87). The mRNA measurements are normalized for library depth, expression frequency across individuals as well as PEER factors, as reported in the GEUVADIS databank^1^. Of the available normalized gene expression phenotypes, only those with non-empty sets of variants lying within 1Mb upstream or downstream of the canonical transcription start site were kept for (cis) fine-mapping. This process yielded 22,664 genes used in all subsequent analyses involving the PICS fine-mapping algorithm.

### 4.1 Probabilistic Identification of Causal SNPs

We implement Probabilistic Identification of Causal SNPs (PICS) (Farh et al. 2015), which is based on an earlier genome-wide analysis identifying genetic and epigenetic maps of causal autoimmune disease variants (Farh et al. 2015).

We assume that X and y are the N×P locus haplotype (or genotype) matrix and the N×1 vector of phenotype values respectively, with y possibly already adjusted by relevant covariates. For consistency of exposition, let the SNPs be named $A_{1},\ldots ,A_{P}$. Recall the goal of fine-mapping is to return information about which SNP(s)in the set $\left\{A_{1},\ldots ,A_{P}\right\}$ is (are) causal, given the input pair {X,y} and possibly other external information such as functional annotations or, more generally, prior biological knowledge.

#### Overview.

Probabilistic Identification of Causal SNPs (PICS) is a Bayesian, non-parametric approach to fine-mapping. Given the observed patterns of association at a locus, and furthermore not assuming a parametric model relating the causal variants to the trait itself, one can estimate the probability that any SNP is causal by performing permutations that preserve its marginal association with the trait as well as the LD patterns at the locus.

To see how this is accomplished, suppose that $A_{1}$ is the lead SNP. We are interested in $P(A_{i}^{\text{causal}}|A_{1}=A^{\text{lead}})$, the probability that $A_{i}$ is causal, for each i∈[P]. By Bayes’ theorem,

(1) $$P(A_{i}^{\text{causal}}|A_{1}=A^{\text{lead}})\propto P(A_{1}=A^{\text{lead}}|A_{i}^{\text{causal}})\times P(A_{i}^{\text{causal}}).$$

**Figure 5:**
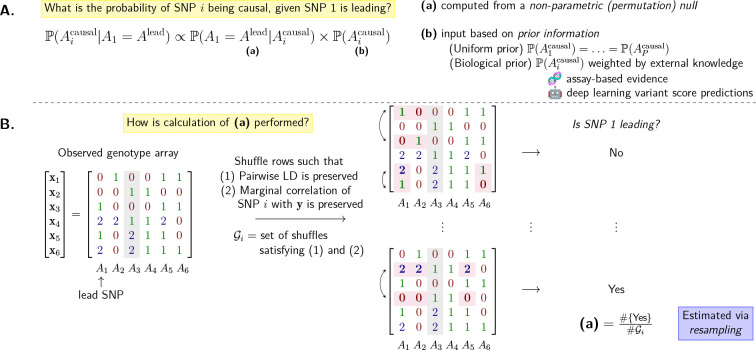
Visual summary of the PICS algorithm described in Subsection 4.1. **A.** Breakdown of the calculation of the probability of a focal SNP $A_{i}$ being causal. **B.** Illustration of the permutation procedure used to generate the null distribution. An example N×P genotype array with N=P=6 is used, with two valid row shuffles, or permutations, of the original array shown. Entries affected by the shuffle are highlighted, as is the focal $SNP\left(A_{3}\right)$.

Focusing on the focal SNP i, permute the rows of X such that the association between the focal SNP and the trait y is invariant; see Supplementary Material Section S1 for a concrete mathematical description of this set of constrained permutations. Then the first term on RHS of eq. (1), $\left({PA_{1}=\;A}^{\text{lead}}|A_{i}^{\text{causal}}\right)$, is estimated by the proportion of all permutations where $A_{1}$ emerges as the lead SNP. The second term, $P(A_{i}^{\text{causal}})$, is the prior probability of the focal SNP being causal, which the user can choose based on prior knowledge. The default setting in PICS is $P(A_{1}^{\text{causal}})=\ldots =P(A_{P}^{\text{causal}})$. Figure 5 provides a visual summary of the method.

By running the permutation procedure across all SNPs in the locus, one obtains $P(A_{i}^{\text{causal}}|A_{1}=A^{\text{lead}})$ for each i. These posterior probabilities are then normalized so that $\sum_{i=1}^{N}\;P(A_{i}^{\text{causal}}|A_{1}=A^{\text{lead}}=1.)$

Finally, the above procedure is performed after restricting the set of all SNPs to only those with correlation magnitude |r|>0.5 to the lead SNP.

#### Algorithm.

Based on the computation of posterior probabilities described above (eq. (1)), the full PICS algorithm returns putatively causal variants as follows.

**Algorithm 1:** PICS

1:	**Input**: Individual-by-genotype array $\underset{N\times P}{X}$, phenotype array $\underset{N\times 1}{y}$, LD threshold |r|, resampling number R, number of potential sets C, prior causal probabilities $\underset{P\times 1}{p_{0}}$.
2:	c←1
3:	**while** c⩽C **do**
4:	M←No. columns of X
5:	Identify lead SNP, ℓ∈{1,…,M}
6:	Identify neighbouring SNPs, ${𝒩}_{\ell }^{c}=\{k\in [M]:cor{\left(A_{k},A_{\ell }\right)}^{2}>r^{2}\}$.
7:	**for** $j\in {𝒩}_{\ell }^{c}$ **do**
8:	Compute $\hat{p_{j}}=P(A_{j}^{\text{causal}\;}|A_{\ell }=A^{\text{lead}\;}))$ as described below eq. (1). Use R permutations.
9:	**end for**
10:	Record vector of posterior probabilities, $\vec{p^{c}}=\left[\hat{p_{j}}:j\in {𝒩}_{\ell }^{c}\right]$
11:	$X\leftarrow X\left[:,\{1,\ldots ,M\}\setminus {𝒩}_{\ell }^{c}\right]$
12:	c←c+1
13:	**end while**
14:	**Output:** $\left[\vec{p^{c}}:c⩽C\right]$ (a list, where component c corresponds to Potential Set c)

The last line of Algorithm 1 returns a list of posterior probability vectors corresponding to each potential set. For our work, we set |r|=0.5, C=3, R=500 and $p_{0}=(1/P,\ldots ,1/P)$ throughout implementations of Algorithm 1.

As mentioned in the Introduction (Section 1), we apply two different approaches to implementing Algorithm 1. One is guided by stability and will be introduced shortly in Subsection 4.2. The other, which we now describe, is based on regressing out confounders, i.e., residualization. In implementing the residualization approach, we regress the top five principal components, obtained from the genotype matrix X, from the gene expression phenotype, y. We choose five principal components based on the elbow method, an approach described in Brown et al. (2018). This yields residuals $y^{r}$ (Figure 1A), which we use as the input phenotype array in Algorithm 1. We subsequently report the variant with highest posterior probability in each potential set as the putatively causal variant for that potential set. This variant is referred to as the top variant.

We remark that there are other ways of using potential confounders in variable selection, such as inclusion as covariates in a linear model. Because our algorithm explicitly avoids assuming a linear model, we have chosen the residualization approach just described.

### 4.2 Incorporating Stability

Our stability-guided approach to implementing PICS follows the steps outlined below. Let (X,y) be the genotype array and gene expression array. Assume that there are K subpopulations making up the dataset, and let $E_{k}$ denote the set of row indices of X corresponding to individuals from subpopulation k. (In our present work, K=5. The five subpopulations are Utahns $\left(N_{CEU}=89\right)$, Finns $\left(N_{FIN}=92\right)$, British $\left(N_{GBR}=86\right)$, Toscani $\left(N_{TSI}=91\right)$ and Yoruban $\left(N_{YRI}=87\right)$.)

1. Run Algorithm 1 on the pair (X,y). Obtain list of posterior probability vectors, $ℒ=[\vec{p}\;:c⩽C]$.
2. For each subpopulation k=1,…,K, nun Algorithm 1 on the pair $\left.{\left.(X\right|}_{E_{k}},y|E_{k}\right)$. Obtain ${ℒ}^{\left(k\right)}=\left[\vec{p_{k}^{c}}:c⩽C\right]$ for each k.
3. (Stability-guided choice of putatively causal variants) Collect the probability vectors in lists ℒ and ${ℒ}^{\left(k\right)}(k\in [K])$. Operationalizing the principle that a stable variant has positive probability across multiple slices, we pick causal variants as follows. For potential set c, pick the variants that have (i) positive probability in $\vec{p^{c}}$ in Step 1, and moreover (ii) have positive probability in the most number of probability vectors; call this set of variants ${𝒮}_{c}$ (note ${𝒮}_{c}$ is a subset of $\vec{p^{c}}$). Among members of ${𝒮}_{c}$, select the variant that had the highest posterior probability in $\vec{p^{c}}$.

In other words, the stability-guided approach reports the variant that not only appears with positive probability in the most number of subsets including the pooled sample, but also has the largest probability in the pooled sample. This variant is referred to as the stable variant.

To illustrate the last step, suppose there are K=2 subpopulations, C=1 potential set, and P=5 SNPs. Let the outputs from Steps 1 and 2 be

$$ℒ:\vec{p^{1}}=(0.45,\;0.43,\;0.02,\;0.10,\;0.00)\;{ℒ}^{\left(1\right)}:\vec{p_{1}^{1}}=(0.00,\;0.40,\;0.10,\;0.25,\;0.25)\;{ℒ}^{\left(2\right)}:\vec{p_{2}^{1}}=(0.20,\;0.60,\;0.00,\;0.10,\;0.10)$$

In this example, the second and fourth variants have positive probability in the most number of probability vectors $\left(S_{1}=\left\{A_{2},A_{4}\right\}\right)$. Among $A_{2}$ and $A_{4},$ $A_{2}$ has a higher posterior probability (i.e., 0.43) in $\vec{p^{1}}$, so the stable variant reported is $A_{2}$. As a side remark, the posterior probability vector ℒ does not generally agree with the posterior probability vector computed using the residualization approach, because the latter is computed by running Algorithm 1 on the pair $\left.{\left(X\right.,y}^{r}\right)$ rather than (X,y), as described earlier.

### 4.3 Statistical Comparison Methodology

We rely on 378 external functional annotations to interpret biological significance of our variants, summarized in Table 1. Supplementary Material Section S2 provides interpretation for the functional annotations, while Supplementary Material Section S3 describes in greater detail how we generate annotations from Enformer predictions.

Our comparison of the top and stable variants is two-fold. First, we evaluate the relative significance of the stable variant against the top variant, by running paired Wilcoxon two-sample tests across all pairs of top and stable variants across all GEUVADIS gene expression phenotypes. We compute one-directional *p*-values in either direction to check for significant depletion or enrichment of the stable variant with respect to a particular annotation. Raw *p*-values are adjusted for false discovery rate control by applying the standard Benjamini-Hochberg (BH) procedure (R command p.adjust(…,method=‘BH’))) to *p*-values across all 378 annotations and all potential sets.

To investigate whether various external factors might moderate the differences in functional annotations, we next perform trend tests. Basically, for some external factor F, we run a trend test to see if values of F are associated with attenuation or augmentation of differences in functional annotation of the top and stable variants. The list of all external factors F is provided in Table 2.

For (1) Degree of Stability, we perform a Jonckheere-Terpstra test (R command clinfun:: jonckheere.test (…, nperm=5000)). For (2) Population Diversity, (3) Population Differentiation and (6) Degree of Certainty of Causality Using Residualization Approach, we perform a correlation test (R command cor.test (…)). For (4) Inclusion of Distal Subpopulations (Top) and (5) Inclusion of Distal Subpopulations (Stable), we perform an unpaired Wilcoxon test (R command wilcox.test(…)). Finally, for each moderator, we again compute one-directional *p*-values in either direction, before applying the BH procedure to all *p*-values across annotations and potential sets for that moderator only.

## Supplementary Material

Supplement 1

## Figures and Tables

**Table 1:** A list of 378 functional annotations across which the biological significances of stable and top fine-mapped single nucleotide polymorphisms are compared.

Functional Annotation Type	Functional Annotation
Ensembl	Distance to Canonical *TSS* (Cunningham et al. 2022) Regulatory Features (6; Cunningham et al. 2022)
Computational Predictions	CADD* (2; Rentzsch et al. 2019) SIFTVal* (Ng and Henikoff 2003) FATHMM-XF* (Rogers et al. 2018) LINSIGHT* (Huang et al. 2017) Polyphen* (Adzhubei et al. 2010) PhyloP* (3; Pollard et al. 2010) Gerp* (2; Davydov et al. 2010) B Statistic* (McVicker et al. 2009) FunSeq2* (Fu et al. 2014) ALoFT* (Balasubramanian et al. 2017) Percent CpG in 75*bp* window* (Rentzsch et al. 2019) Percent GC in 75*bp* window* (Rentzsch et al. 2019) FIRE (Ioannidis et al. 2017) Enfermer (177 tracks × 2 scores per track; Avsec et al. 2021)

Annotations that report multiple scores have the total number of scores reported shown in parentheses. Scores mined from the FAVOR database (Zhou et al. 2022) are indicated by an asterisk. (*TSS* = Transcription Start Site, *bp* = base pair)

**Table 2:** List of 6 moderating factors considered.

Moderator	Quantity/Statistic Computed
(1) Degree of Stability	No. subpopulations for which stable variant has positive probability
(2) Population Diversity	Maximum of pairwise allele frequency difference between subpopulations for which stable variant has positive posterior probability
(3) Population Differentiation	Maximum *F_ST_* between subpopulations for which stable variant has positive posterior probability
(4) Inclusion of Distal Subpopulations (Top)	Whether or not the top variant also had positive probability in Yoruban subpopulation when the stability-guided approach was used
(5) Inclusion of Distal Subpopulations (Stable)	Whether or not the stable variant had positive probability in Yoruban subpopulation when the stability-guided approach was used
(6) Degree of Certainty of Causality Using Residualization Approach	Posterior probability of top variant

## Data Availability

We provide the following data and scripts on the Github repository https://github.com/songlab-cal/StableFM: fine-mapped variants with their functional annotations and moderator variable quantities, code for reproducing figures in this manuscript and building our visualization app.

## Footnotes

1
https://www.ebi.ac.uk/biostudies/files/E-GEUV-1/E-GEUV-1/analysis_results/GeuvadisRNASeqAnalysisFiles_README.txt
