# Supplementary material for: The Impact of Stability Considerations on Genetic Fine-Mapping
Source: bioRxiv. 2023 Apr 13:2023.04.11.536456. Preprint. [Version 1] doi: 10.1101/2023.04.11.536456 (PMC10120703; doi:10.1101/2023.04.11.536456)
Supplement: Supplement 1 [file NIHPP2023.04.11.536456v1-supplement-1.pdf]

bioRxiv preprint doi: https://doi.org/10.1101/2023.04.11.536456; this version posted April 13, 2023. The copyright holder for this preprint (which was not certified by peer review) is the author/funder, who has granted bioRxiv a license to display the preprint in perpetuity. It is made available under aCC-BY 4.0 International license.

Supplementary Material
The Impact of Stability Considerations on Genetic Fine-Mapping

Alan Aw[1,2], Lionel Chentian Jin[3], Nilah Ioannidis[2,4,*], Yun S. Song[1,2,4,*]

[1] Department of Statistics, University of California, Berkeley
[2] Center for Computational Biology, University of California, Berkeley
[3] McKinsey & Company, Seattle
[4] Computer Science Division, University of California, Berkeley

March 23, 2023

S1 Permuting while preserving marginal correlation and LD: a Formal Description

For a focal SNP i, its N entries $(x_{1i}, \ldots, x_{Ni})$ take on only D values, where D is the ploidy of the data (for human genotypes $D = 2$ and for human haplotypes $D = 1$). Suppose $D = 2$ for exposition. For $d = 0, 1, 2$, let $I_d \subset [N]$ denote the indices of those elements of the allelic dosage vector $(x_{1i}, \ldots, x_{Ni})$ taking on value d. Then, any permutation $\sigma \in \mathfrak{S}_N$ of the rows of $\mathbf{X}$ that preserves marginal correlation with the phenotype and LD maps indices from I_d to I_d, because

- Each distinct entry in the allelic dosage vector of the focal SNP is still assigned the the same phenotype entry;
- Row shuffles do not change column-column covariances, the latter of which determines LD.

To be precise, $\sigma \in \mathfrak{S}_{|I_0|} \times \mathfrak{S}_{|I_1|} \times \mathfrak{S}_{|I_2|}$, a direct product of the three permutation groups corresponding to the different allelic dosages.

In practice, we approximate the permutation distribution by sampling permutations uniformly at random from the group defined above. We set the sampling number to 500 for all our fine-mapping experiments.

S2 List of Annotations

We measure the biological significance of a variant using a wide range of available functional annotations (Table S1). The annotations cover potential biological activity in the local vicinity of the variant position along the genome, estimated model-based biological quantities (e.g., selection and conservation scores), and predicted effects of mutagenesis from the reference to the alternate allele at the variant.

*To whom correspondence should be addressed: nilah@berkeley.edu, yss@berkeley.edu

bioRxiv preprint doi: https://doi.org/10.1101/2023.04.11.536456; this version posted April 13, 2023. The copyright holder for this preprint (which was not certified by peer review) is the author/funder, who has granted bioRxiv a license to display the preprint in perpetuity. It is made available under aCC-BY 4.0 International license.

Table S1: List of annotations used in our study, alongside their interpretations.

Functional Annotation	Interpretation
Distance to Canonical Transcription Start Site (TSS)	-
Percent CpG in 75 bp window centered on variant position	-
Percent GC in 75 bp window centered on variant position	-
CTCF Binding Enrichment	whether the variant lies within a CTCF binding site region as predicted by Ensembl
Enhancer Enrichment	whether the variant lies within an enhancer region as predicted by Ensembl
Open Chromatin Enrichment	whether the variant lies within an open chromatin region as predicted by Ensembl
Promoter Enrichment	whether the variant lies within a promoter region as predicted by Ensembl
TF Binding Enrichment	whether the variant lies within a TF binding site region as predicted by Ensembl
Promoter Flanking Enrichment	whether the variant lies within a promoter flanking region as predicted by Ensembl
CADD (2 scores)	whether the variant is likely to be simulated or not, and hence likely deleterious or not. One score is raw while the other is rank-normalized
SIFTVal	whether the variant affects protein function, and hence deleterious
Polyphen2	posterior probability that the variant is damaging
LINSIGHT	probability that the variant site is under selection, thus having functional consequence
PhyloP (3 scores)	substitution rates measuring cross-species evolutionary conservation at the site of the variant. Each score is computed with respect to a clade (vertebrate, mammal, primate)
GerpN	estimated neutral substitution rate at variant position, with higher value implying greater conservation
GerpS	estimated rejected substitution rate at variant position, with positive value implying a deficit in substitutions
B Statistic	background selection at variant position, with smaller value indicating larger impact of selection
FATHMM-XF	integrative score measuring deleteriousness of the variant
Funseq2	integrative score measuring deleteriousness of the variant
ALoft	integrative score measuring loss of function associated with the variant
FIRE	integrative score measuring deleteriousness of the variant
Magnitude of Effect on Enformer Track Prediction (177 tracks)	change in prediction of a gene regulatory track when performing in-silico mutagenesis on the variant in a 196,608 bp sequence

bioRxiv preprint doi: https://doi.org/10.1101/2023.04.11.536456; this version posted April 13, 2023. The copyright holder for this preprint (which was not certified by peer review) is the author/funder, who has granted bioRxiv a license to display the preprint in perpetuity. It is made available under aCC-BY 4.0 International license.

S3 Generating Annotations from Enformer Predictions

The Enformer (Avsec et al., 2021) is a sequence-based prediction model, which leverages the attention mechanism in a transformer neural network to capture long-range effects on gene regulation.

For Enformer predictions, we subset the 5,313 ChIP-seq, DNase-seq, ATAC-seq and CAGE tracks to only those relevant to the lymphoblastoid cell line (GM12878), the cell line with respect to which GEUVADIS gene expression phenotypes are measured. We also restrict to genes whose top and stable variants are within the 196,608 bp input length limit of the Enformer from the corresponding GEUVADIS gene's TSS. This restriction allows us to obtain Enformer predictions on three sequences: a *null sequence*, a sequence where the REF allele is replaced with the ALT allele at the top variant (*top sequence*), and a sequence where the REF allele is replaced with the ALT allele at the stable variant (*stable sequence*).

To compare the top and the stable variants with respect to a particular track, we take the predictions obtained from the three input sequences (null prediction, top prediction, stable prediction — a *triplet*), and compute the magnitude of change in predictions between both the top and null and the stable and null. We use the magnitude of change here, rather than the change itself, to capture the impact the mutation associated with the variant has on the track. Our Enformer functional annotations can thus be interpreted as measures of mutagenic impact on a track's prediction, without considering the directionality of the impact. We refer to these annotations as *perturbation scores*.

We compute perturbation scores in two ways. The first way is to center all input sequences on the transcription start site of the gene (occasionally referred to as 'TSS' in our work), thus allowing the model to measure the impact of mutagenesis at the (top or stable) variant on predicted gene expression profile for the sequence. The second way is to average the perturbation scores over three triplets, one centered on the gene TSS, and two centered on the flanking positions of the gene TSS (i.e., the two neighbouring bins). The second way (occasionally referred to as 'AVE' in our work) accounts for errors arising from imprecise TSS positioning and the instability of the model to small changes in input — a technique used in Karollus et al. (2022) and by the Enformer team (Avsec et al., 2021).

S4 Matching versus Non-matching Variant Results

As described in the main text, we compare between one set of genes where the top and stable variants match and another set of genes where the top and stable variants disagree; comparisons are made between matching variants and one of the non-matching sets of variants (top or stable).

Concretely, for Potential Set 1 we compare between $N_{\text{match}} = 12,743$ genes with matching variants and $N_{\text{non-match}} = 9,921$ genes with non-matching variants; for Potential Set 2 we compare between $N_{\text{match}} = 8,197$ genes with matching variants and $N_{\text{non-match}} = 14,452$ genes with non-matching variants; and for Potential Set 3 we compare between $N_{\text{match}} = 5,807$ genes with matching variants and $N_{\text{non-match}} = 16,812$ genes with non-matching variants. Below, we summarize our findings.

Matching Variants vs Non-matching Top Variants. We observe the following significant trends: as mentioned in the main text, for Potential Set 1 361 functional annotations reported significantly higher scores for the matching variants, with all BH-adjusted p-values less than 0.05. The remaining $17 = 378 - 361$ annotations *not* exhibiting significantly higher scores are: Distance to Canonical TSS, CTCF Binding Enrichment, Enhancer Enrichment, Open Chromatin Enrichment, TF Binding Enrichment, Promoter Flanking Enrichment, CADD raw score, PHRED-normmalized

bioRxiv preprint doi: https://doi.org/10.1101/2023.04.11.536456; this version posted April 13, 2023. The copyright holder for this preprint (which was not certified by peer review) is the author/funder, who has granted bioRxiv a license to display the preprint in perpetuity. It is made available under aCC-BY 4.0 International license.

CADD score, SIFTVal, priPhyloP, mamPhyloP, verPhyloP, GerpS, LINSIGHT, Funseq2, ALoft, and FIRE.

For Potential Set 2, 132 functional annotations reported significantly higher scores for the matching variants. For Potential Set 3, 9 functional annotations reported significantly higher scores for the matching variants: B Statistic, percent GC content, and Enformer tracks `ENCFF776DPQ`, `ENCFF831ZHL`, `ENCFF601YET`, `ENCFF945XXY`, `ENCFF676UXN`, `ENCFF151LGF`, `ENCFF876DXW`. For all three potential sets, there were no functional annotations reporting significantly lower scores for the matching variants, even for annotations where a low score implies greater biological functionality (e.g., B Statistic).

Matching Variants vs Non-matching Stable Variants. We observe the following significant trends: as mentioned in the main text, for Potential Set 1 363 functional annotations reported significantly higher scores for the matching variants, with all BH-adjusted p-values less than 0.05. The remaining 15 annotations *not* exhibiting significantly higher scores are: Distance to Canonical TSS, CTCF Binding Enrichment, Enhancer Enrichment, Open Chromatin Enrichment, TF Binding Enrichment, Promoter Flanking Enrichment, CADD raw score, PHRED-normmalized CADD score, SIFTVal, mamPhyloP, verPhyloP, GerpS, LINSIGHT, ALoft, and FIRE.

For Potential Set 2, 359 functional annotations reported significantly higher scores for the matching variants (all BH-adjusted p-values < 0.05), while only one functional annotation — Distance to Canonical TSS — reported significantly lower scores for the matching variants (BH-adjusted p-value $= 8 \times 10^{-4}$). For Potential Set 3, 259 functional annotations reported significantly higher scores for the matching variants (all BH-adjusted p-values < 0.05), while only one functional annotation — Distance to Canonical TSS — reported significantly lower scores for the matching variants (BH-adjusted p-value $= 8 \times 10^{-4}$).

S5 Top Variant versus Stable Variant Results

As described in the main text, we compare, across all genes, the annotations of the top and the stable variant. We reported no significant differences in enrichment or trends in enrichment differences driven by moderators for Potential Set 1. For completeness, we report here significant results for all three potential sets.

Paired analysis. After BH-adjustment, there are no significant differences across all three potential sets using a threshold of $\alpha = 0.05$.

Trend analysis. For the following moderators, we observe some significant trends.

- Inclusion of Distal Subpopulations (Top)

  For Potential Set 3, FIRE scores exhibited a greater difference between stable and top variant scores (i.e., stable score − top score) for genes with top variants not discovered in YRI than those with top variants discovered in YRI (one-sided BH-adjusted $p = 0.03$). So did TSS-centered perturbation scores for 121 Enformer tracks and AVE perturbation scores for 121 Enformer tracks. (These tracks and their corresponding BH-adjusted p-values are available at: `https://github.com/songlab-cal/StableFM/blob/master/data/results_with_moderators/p_values/mod_Top%20SNV%20Discovered%20in%20YRI.csv`.)

- Posterior Probability of Top Variant

bioRxiv preprint doi: https://doi.org/10.1101/2023.04.11.536456; this version posted April 13, 2023. The copyright holder for this preprint (which was not certified by peer review) is the author/funder, who has granted bioRxiv a license to display the preprint in perpetuity. It is made available under aCC-BY 4.0 International license.

For all potential sets, there are small but significant positive correlations between the posterior probability of the top variant and the difference between the stable variant FIRE score and the top variant FIRE score (Potential Set 1: Pearson's $r = 0.05$, BH-adjusted $p = 3 \times 10^{-5}$; Potential Set 2: Pearson's $r = 0.07$, BH-adjusted $p = 3 \times 10^{-14}$; Potential Set 3: Pearson's $r = 0.05$, BH-adjusted $p = 1 \times 10^{-7}$). For all potential sets, there are small but significant negative correlations between the posterior probability of the top variant and the difference between the stable variant Absolute Distance to TSS and the top variant Absolute Distance to TSS (Potential Set 1: Pearson's $r = -0.07$, BH-adjusted $p = 1 \times 10^{-10}$; Potential Set 2: Pearson's $r = -0.05$, BH-adjusted $p = 9 \times 10^{-7}$; Potential Set 3: Pearson's $r = -0.06$, BH-adjusted $p = 2 \times 10^{-12}$). For Potential Set 2, there are significant negative correlations between the posterior probability of the top variant and the difference between the stable variant and top variant Percent GC (Pearson's $r = -0.04$, BH-adjusted $p = 5 \times 10^{-5}$) and FATHMM-XF scores (Pearson's $r = -0.04$, BH-adjusted $p = 7 \times 10^{-4}$). For Potential Set 3, there is a significant negative correlation between the posterior probability of the top variant and the difference between the stable variant and top variant FATHMM-XF scores (Pearson's $r = -0.04$, BH-adjusted $p = 2 \times 10^{-5}$).

S6 Impact of Positive Posterior Probability Support on Results

One key consideration in statistical fine-mapping is the number of variants possessing positive posterior probability, which we refer to as the *positive posterior probability support* (or *support*, for short). Indeed, a larger support may indicate greater uncertainty in the assignment of causal variant to the allele with largest posterior probability. To evaluate the potential utility of the stability-guided approach in settings where the residualization approach leads to large support, we repeat our analysis on two restricted sets of genes, namely (1) those genes where the top variant has support greater than 10; or (2) those genes where the top variant has support greater than 50.

Paired analysis. *Gene Set (1).* For all Potential Sets, Distance to TSS is significantly smaller for top variant (all three BH-adjusted p-values $< 10^{-6}$). For Potential Set 3, FATHMM-XF score is significantly larger for stable variant (BH-adjusted $p < 10^{-6}$). For all Potential Sets, FIRE score is significantly larger for top variant (BH-adjusted p-values: Potential Set 1 = 0.02, Potential Set 2 = 2×10^{-10}, Potential Set 3 = 1.7×10^{-4}). *Gene Set (2).* For all Potential Sets 2 and 3, FIRE score is significantly larger for top variant (both BH-adjusted p-values are 0.04).

Trend analysis.
Gene Set (1). For the following moderators, we observe some significant trends.

- Posterior Probability of Top Variant

  For Potential Sets 2 and 3, there is a significant negative correlation between the posterior probability of the top variant and the difference between the stable variant FIRE score and the top variant FIRE score (Potential Set 2: Pearson's $r = 0.08$, BH-adjusted $p = 1.9 \times 10^{-5}$; Potential Set 3: Pearson's $r = 0.06$, BH-adjusted $p = 0.003$). For Potential Set 2, there is a significant positive correlation between the posterior probability of the top variant and the difference between the stable variant's FATHMM-XF score and the top variant's FATHMM-XF score (Pearson's $r = -0.06$, BH-adjusted $p = 0.01$).

Gene Set (2). For the following moderators, we observe some significant trends.

- Posterior Probability of Top Variant

bioRxiv preprint doi: https://doi.org/10.1101/2023.04.11.536456; this version posted April 13, 2023. The copyright holder for this preprint (which was not certified by peer review) is the author/funder, who has granted bioRxiv a license to display the preprint in perpetuity. It is made available under aCC-BY 4.0 International license.

For Potential Set 2, TSS-centered perturbation scores for 8 Enformer tracks exhibited a positive correlation between the posterior probability of the top variant and the difference between stable and top variant perturbation scores. Track names (with empirical Pearson's r) are ENCFF915DFR (-0.40), ENCFF107LDM (-0.41), ENCFF821PRO (-0.42), ENCFF171MDW (-0.43), ENCFF170NTY (-0.43), ENCFF935KTD (-0.41), ENCFF676GTP (-0.41), ENCFF013ZOI (-0.42). Averaged perturbation scores for two Enformer tracks also exhibited negative correlation; these two tracks are ENCFF107LDM (-0.40) and ENCFF170NTY (-0.40). All BH-adjusted p-values are 0.0495.

S7 Impact of Posterior Probability on Results

We perform a comparison between top and stable variants, by restricting to genes where the posterior probability of the top variant or the stable variant exceeds 0.9. Specifically, we repeat our analysis on two restricted sets of genes, namely (1) those genes where the top variant reported a posterior probability exceeding 0.9; or (2) those genes where the stable variant reported a posterior probability exceeding 0.9. For reference, we plot in Figure S2 the joint distribution of posterior probabilities of top and stable variants, across all genes for which the two fine-mapping approaches returned distinct variants.

Paired analysis. *Gene Set (1).* For Potential Set 3, Distance to TSS is significantly smaller for stable variant (BH-adjusted $p = 0.01$). *Gene Set (2).* For Potential Sets 1 and 2, FIRE scores of the top variant are significantly larger than the stable variant (BH-adjusted p-values: Potential Set $1 = 1 \times 10^{-6}$, Potential Set $2 = 0.02$). For Potential Set 2, Distance to TSS of the top variant is significantly smaller (BH-adjusted $p = 3 \times 10^{-4}$). For Potential Set 3, FATHMM-XF scores of the stable variant are significantly larger (BH-adjusted $p = 0.006$).

Trend analysis.
Gene Set (1). For the following moderators, we observe some significant trends.

- Inclusion of Distal Subpopulations (Top)

  For Potential Set 3, TSS-centered perturbation scores for 9 Enformer tracks exhibited a higher difference between stable and top variant perturbation score for genes with top variants not discovered in YRI versus those discovered in YRI. Track names (with BH-adjusted unpaired Wilcoxon test p-values) are ENCFF279CYY (0.03), ENCFF417WYL (0.04), ENCFF782WWH (0.03), ENCFF629RRF (0.03), ENCFF676GTP (0.03), ENCFF984HLU (0.03), ENCFF700YOH (0.03), ENCFF848LJL (0.0477), ENCFF038IYA (0.03). Averaged perturbation scores for 10 Enformer tracks also a higher difference; these are ENCFF776DPQ (0.02), ENCFF279CYY (0.02), ENCFF629RRF (0.03), ENCFF319YAI (0.04), ENCFF676GTP (0.046), ENCFF367WTF (0.04), ENCFF984HLU (0.04), ENCFF917YSR (0.03), ENCFF613CYH (0.04) and ENCFF700YOH (0.04).

Gene Set (2). For the following moderators, we observe some significant trends.

- Posterior Probability of Top Variant

  For Potential Set 3, there is a significant negative correlation between the posterior probability of the top variant and the difference between the stable variant Distance to TSS and the top variant Distance to TSS (Pearson's $r = -0.09$, BH-adjusted $p = 0.004$).

bioRxiv preprint doi: https://doi.org/10.1101/2023.04.11.536456; this version posted April 13, 2023. The copyright holder for this preprint (which was not certified by peer review) is the author/funder, who has granted bioRxiv a license to display the preprint in perpetuity. It is made available under aCC-BY 4.0 International license.

References

Žiga Avsec, Vikram Agarwal, Daniel Visentin, Joseph R Ledsam, Agnieszka Grabska-Barwinska, Kyle R Taylor, Yannis Assael, John Jumper, Pushmeet Kohli, and David R Kelley. Effective gene expression prediction from sequence by integrating long-range interactions. *Nature Methods*, 18 (10):1196–1203, 2021.

Alexander Karollus, Thomas Mauermeier, and Julien Gagneur. Current sequence-based models capture gene expression determinants in promoters but mostly ignore distal enhancers. *bioRxiv*, 2022.

bioRxiv preprint doi: https://doi.org/10.1101/2023.04.11.536456; this version posted April 13, 2023. The copyright holder for this preprint (which was not certified by peer review) is the author/funder, who has granted bioRxiv a license to display the preprint in perpetuity. It is made available under aCC-BY 4.0 International license.

Supplementary Figures

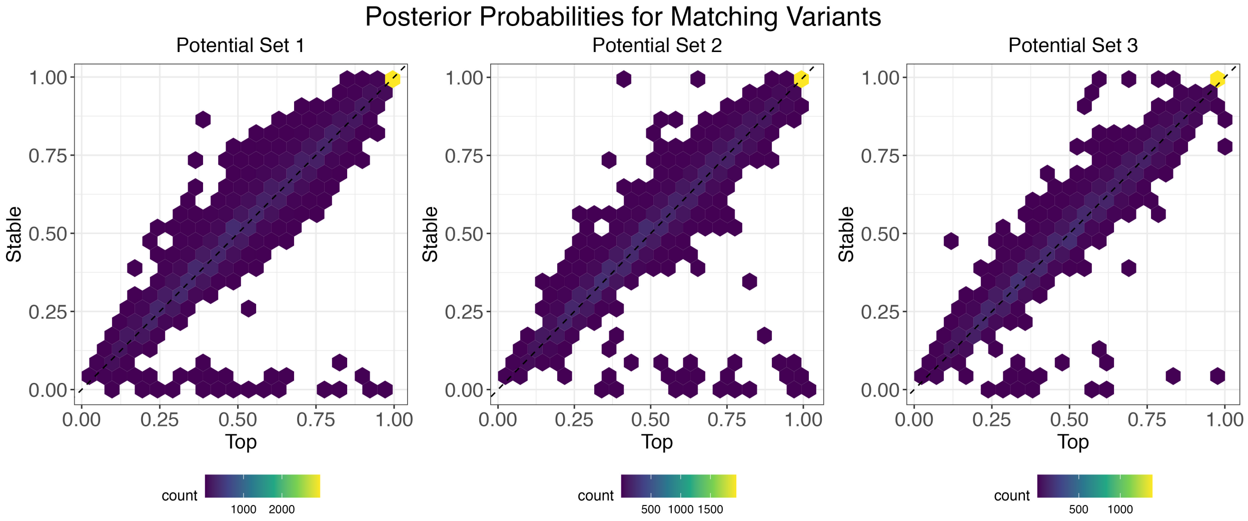

Figure S1: Pair density plot of posterior probabilities of the top variant and the stable variant, in case they match.

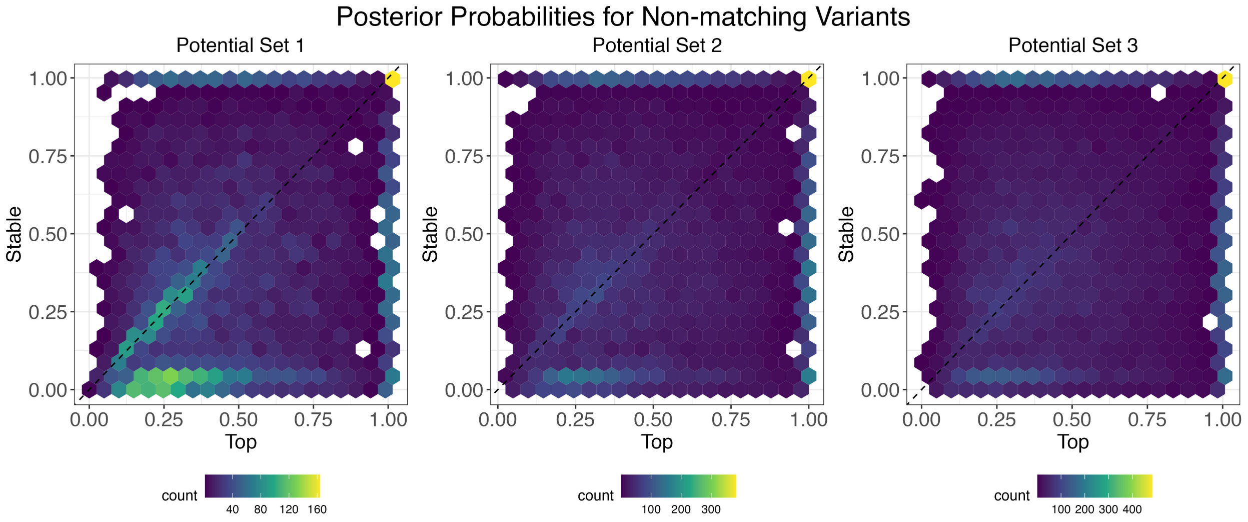

Figure S2: Pair density plot of posterior probabilities of the top variant and the stable variant, in case they do not match.
